# Supplementary material for: HIVprotI: an integrated web based platform for prediction and design of HIV proteins inhibitors
Source: J Cheminform. 2018 Mar 9;10:12. doi: 10.1186/s13321-018-0266-y (PMC5845081; doi:10.1186/s13321-018-0266-y)
Supplement: Supplementary file 2 — Additional file 2. Source code of HIVProtI web server. [file 13321_2018_266_MOESM2_ESM.zip › HIVprotI_Source-code/hivprotI/weka-3-6-6/doc/weka/classifiers/bayes/blr/Prior.html]

Prior


---


|  |  |  |
| --- | --- | --- |
| **Overview** \| **Package** \| **Class** \| **Tree** \| **Deprecated** \| **Index** \| **Help** \| **Weka's home** | |  |
| **PREV CLASS**   NEXT CLASS | **FRAMES**    **NO FRAMES**     **All Classes** |  |
| SUMMARY: NESTED \| FIELD \| CONSTR \| METHOD | DETAIL: FIELD \| CONSTR \| METHOD |  |


---


## weka.classifiers.bayes.blr Class Prior

```
java.lang.Object
  weka.classifiers.bayes.blr.Prior
```

**All Implemented Interfaces:**: java.io.Serializable, RevisionHandler

**Direct Known Subclasses:**: GaussianPriorImpl, LaplacePriorImpl

---

``` public abstract class Prior extends java.lang.Object implements java.io.Serializable, RevisionHandler ```

This is an interface to plug various priors into
the Bayesian Logistic Regression Model.

**Version:**
:   $Revision: 1.2 $

**Author:**
:   Navendu Garg (gargnav@iit.edu)

**See Also:**: Serialized Form

---

**Constructor Summary**

`Prior()`


| **Method Summary** | |
| --- | --- |
| `void` | `computelogLikelihood(double[] betas, Instances instances)`             Function computes the log-likelihood value: -sum{1 to n}{ln(1+exp(-Beta\*x(i)\*y(i))} |
| `void` | `computePenalty(double[] betas, double[] hyperparameters)`             Skeleton function to compute penalty terms. |
| `double` | `getLoglikelihood()` |
| `double` | `getLogPosterior()` |
| `double` | `getPenalty()` |
| `double` | `update(int j, Instances instances, double beta, double hyperparameter, double[] r, double deltaV)`             Interface for the update functions for different types of priors. |

**Methods inherited from class java.lang.Object**

`equals, getClass, hashCode, notify, notifyAll, toString, wait, wait, wait`

**Methods inherited from interface weka.core.RevisionHandler**

`getRevision`

**Constructor Detail**

### Prior

```
public Prior()
```


**Method Detail**

### update

```
public double update(int j,
                     Instances instances,
                     double beta,
                     double hyperparameter,
                     double[] r,
                     double deltaV)
```

:   Interface for the update functions for different types of
    priors.

---


### computelogLikelihood

```
public void computelogLikelihood(double[] betas,
                                 Instances instances)
```

:   Function computes the log-likelihood value:
    -sum{1 to n}{ln(1+exp(-Beta\*x(i)\*y(i))}

    :   **Parameters:**: `betas` -: `instances` -

---


### computePenalty

```
public void computePenalty(double[] betas,
                           double[] hyperparameters)
```

:   Skeleton function to compute penalty terms.

    :   **Parameters:**: `betas` -: `hyperparameters` -

---


### getLoglikelihood

```
public double getLoglikelihood()
```

:   **Returns:**: log-likelihood value.

---


### getLogPosterior

```
public double getLogPosterior()
```

:   **Returns:**: regularized log posterior value.

---


### getPenalty

```
public double getPenalty()
```

:   **Returns:**: penalty term.


---


|  |  |  |
| --- | --- | --- |
| **Overview** \| **Package** \| **Class** \| **Tree** \| **Deprecated** \| **Index** \| **Help** \| **Weka's home** | |  |
| **PREV CLASS**   NEXT CLASS | **FRAMES**    **NO FRAMES**     **All Classes** |  |
| SUMMARY: NESTED \| FIELD \| CONSTR \| METHOD | DETAIL: FIELD \| CONSTR \| METHOD |  |


---
